# CORRELATES OF MILD BEHAVIORAL IMPAIRMENT IN OLDER ADULTS WITH COGNITIVE DECLINE

**DOI:** 10.1093/geroni/igad104.3247

**Published:** 2023-12-21

**Authors:** Seolah Yoon, Innhee Jeong, Jennifer (Jeehyun) Kim, Bada Kang

**Affiliations:** Yonsei University College of Nursing, Seoul, Republic of Korea; Yonsei University College of Nursing, Seoul, Republic of Korea; Yonsei University College of Nursing, Seoul, Republic of Korea; Yonsei University College of Nursing, Seoul, Republic of Korea

## Abstract

Implementing early interventions in individuals with subjective cognitive decline and mild cognitive impairment during preclinical and prodromal stage of Alzheimer’s dementia spectrum, addresses modifiable risk factors thereby reducing potential dementia. Mild behavioral impairment (MBI) is considered as a potential predictor and prognostic marker for dementia. However, comprehensive reviews on how MBI is associated with other factors in these at-risk groups are limited. This scoping review identified and mapped associated factors of MBI on health outcomes among older adults with subjective cognitive decline and mild cognitive impairment. Following Joanna Briggs Institute methodology, we sourced studies from databases including PubMed (MEDLINE), CINAHL, Web of Science, EMBASE, PsycINFO, Cochrane, and SCOPUS, focusing on English-language publications published between January 2003 and July 2023. A total of 36 studies met the inclusion criteria. We classified MBI correlates identified from these studies into three categories: neurocognitive, physical, and psychosocial. Neurocognitive functions such as objective cognitive decline and progression to Alzheimer’s disease showed distinct health outcomes related to the MBI. Biomarkers including brain atrophy, beta-amyloid, and p-tau were found to be associated with MBI. Frailty, low gait speed, and hearing loss were physical functional outcomes associated with MBI. Studies on psychosocial aspects with MBI were limited, except one study demonstrating the relationship between caregiver burdens and MBI severity. This review underscores the importance of taking into account MBI and its correlates for developing tailored care approaches to prevent functional decline and improving health outcomes among older adults with subjective cognitive decline and mild cognitive impairment.

